# StopKB: a comprehensive knowledgebase for nonsense suppression therapies

**DOI:** 10.1093/database/baae108

**Published:** 2024-10-12

**Authors:** Nicolas Haas, Julie Dawn Thompson, Jean-Paul Renaud, Kirsley Chennen, Olivier Poch

**Affiliations:** Complex Systems and Translational Bioinformatics (CSTB), ICube laboratory—CNRS, University of Strasbourg, CRBS, 1 rue Eugène Boeckel, Strasbourg 67000, France; Complex Systems and Translational Bioinformatics (CSTB), ICube laboratory—CNRS, University of Strasbourg, CRBS, 1 rue Eugène Boeckel, Strasbourg 67000, France; Urania Therapeutics, 15 rue Neuve, Ostwald 67540, France; Complex Systems and Translational Bioinformatics (CSTB), ICube laboratory—CNRS, University of Strasbourg, CRBS, 1 rue Eugène Boeckel, Strasbourg 67000, France; Complex Systems and Translational Bioinformatics (CSTB), ICube laboratory—CNRS, University of Strasbourg, CRBS, 1 rue Eugène Boeckel, Strasbourg 67000, France

## Abstract

Nonsense variations, characterized by premature termination codons, play a major role in human genetic diseases as well as in cancer susceptibility. Despite their high prevalence, effective therapeutic strategies targeting premature termination codons remain a challenge. To understand and explore the intricate mechanisms involved, we developed StopKB, a comprehensive knowledgebase aggregating data from multiple sources on nonsense variations, associated genes, diseases, and phenotypes. StopKB identifies 637 317 unique nonsense variations, distributed across 18 022 human genes and linked to 3206 diseases and 7765 phenotypes. Notably, ∼32% of these variations are classified as nonsense-mediated mRNA decay-insensitive, potentially representing suitable targets for nonsense suppression therapies. We also provide an interactive web interface to facilitate efficient and intuitive data exploration, enabling researchers and clinicians to navigate the complex landscape of nonsense variations. StopKB represents a valuable resource for advancing research in precision medicine and more specifically, the development of targeted therapeutic interventions for genetic diseases associated with nonsense variations.

**Database URL**: https://lbgi.fr/stopkb/

## Introduction

Nonsense variations are responsible for ∼12% of rare human genetic diseases [1] and have been observed in ∼10% of tumor suppressor genes, including the *TP53* gene that is involved in more than 50% of human cancers [2]. A nonsense variation is defined as the change of a nucleotide in the protein-coding sequence of a gene that leads to the appearance of one of the three (UAA, UGA, or UAG) premature termination codons (PTCs) in the mRNA, thus inducing the premature termination of the protein synthesis. During translation, an mRNA with a PTC has two possible fates: (i) either the mRNA is detected and degraded by the cellular surveillance pathway called nonsense-mediated mRNA decay (NMD) [3–5], or (ii) the mRNA escapes the NMD degradation pathway and produces a truncated protein that may have deleterious effects on the cell and the organism. In both cases, the full-length protein is not produced, which is the main cause of the pathology.

PTCs are present in a large range of genetic diseases and cancers, and the development of effective therapeutic solutions (called nonsense suppression therapies) is a major challenge [6]. A diverse array of strategies has emerged that aim to suppress translation termination at in-frame PTCs. For example, nucleic acid-based approaches have shown promising results, encompassing antisense oligonucleotides, suppressor tRNAs, ADAR-catalyzed RNA editing, RNA pseudouridylation, and the revolutionary CRISPR technology for direct genome editing [7]. Small molecule therapies also exist, such as aminoglycoside antibiotics and ataluren [8], which aim to induce translational readthrough (RT) of PTCs. RT is a natural mechanism that can override premature translation termination. In fact, when the eukaryotic ribosome terminates the translation of an mRNA, a stop codon enters the A site of the ribosome, and a competition begins between the translation termination factors and the aminoacyl-tRNAs responsible for the elongation of the polypeptide. Since there are no tRNAs with anticodons complementary to stop codons, the termination factors bind to the stop codon with higher affinity, resulting in a relatively accurate and efficient translation termination [7]. Nevertheless, this process is not infallible, and RT can take over. During RT, a near-cognate tRNA (whose anticodon is complementary to two of the three nucleotides of the stop codon) competes with termination factors at the A site of the ribosome, leading to misreading of the stop codon, insertion of an amino acid [9], and prolonged translation until the ribosome encounters the natural stop codon. It has been estimated that a natural RT mechanism occurs with a frequency of <0.1% for natural stop codons, while it can be as high as 1% for PTCs [9]. Currently, the most promising therapeutic approaches are RT-inducing molecules that partially restore full-length functional protein synthesis [10].

Even so, restoration of the functional protein was only observed for some nonsense variations and for a small portion of the tested cohorts [11]. Moreover, the toxicity of these approaches hinders their consideration as long-term treatments. Thus, the development of new therapies remains necessary for long-term administration, which is inherent to the chronic treatment of a genetic disease.

Several critical factors should be taken into account, in the study of therapeutic strategies for nonsense mutations. These include the presence of the NMD mechanism, which can influence the effectiveness of such therapies [10]. In fact, the position of the PTC within the mRNA is crucial, as its location will determine whether the NMD mechanism is triggered or not. If the PTC is situated less than 150 nucleotides downstream from the start codon, less than 50 nucleotides upstream of the last exon–exon junction, or within the last exon, the NMD mechanism will not be activated [12]. Additionally, the minor allele frequency (MAF), which refers to the frequency at which the second most prevalent allele appears in a population, of nonsense variations across global populations and within specific ethnic groups highlights the genetic diversity and its pathogenicity. Other factors such as the identity of the PTC and the nucleotide context have been shown to affect the efficiency of RT and the identity of the amino acid restored in place of the PTC [11, 13]. Indeed, it has been reported [14, 15] that in the context of natural or gentamicin-induced RT, RT rates were higher for the UGA codon, followed by UAG and then UAA. Furthermore, the nucleotide immediately following the stop codon appears to exert a significant influence on RT, the C nucleotide being the most favorable. Other nucleotides further from the stop codon also seem to have a subtle yet discernible impact on RT [16].

The information required to address these different factors is scattered across multiple databases, and is difficult to access for researchers and clinicians studying nonsense variations and diseases suitable for nonsense suppression therapies. Therefore, we have developed a public knowledgebase named StopKB. StopKB aims to bring together all known nonsense variations and their associated genes, diseases and phenotypes, combined with NMD sensitivity, nucleotide context and MAF data to facilitate the analysis, evaluation and prioritization of PTCs and associated diseases.

## Material and methods

### Data processing

**Figure 1.**
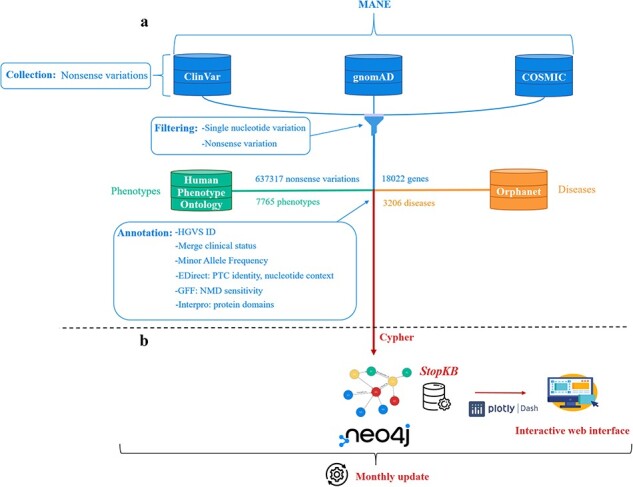
StopKB workflow. (a) Nonsense variations and genes are collected and filtered from three main data sources: ClinVar, gnomAD, and COSMIC. Links to diseases and related phenotypes are retrieved from Orphanet and HPO, respectively. (b) The data are integrated in a graph knowledge base using Neo4j, and are accessible through an interactive web interface. StopKB is updated monthly.

The construction of the dataset for StopKB involves three key steps: data collection, filtering, and annotation (Fig. 1a).

### Data collection

In the initial version of StopKB, we collected data from multiple sources. First, we extracted the nonsense variations from ClinVar (latest release, 2024–04), COSMIC (v99) and gnomAD (v4.0.0) [17–19], with their coordinates corresponding to the latest version of the human genome (GRCh38). We used the Matched Annotation from NCBI and EMBL-EBI (MANE) [20] project as a source to match transcripts and proteins from the ENSEMBL [21] database (which COSMIC and gnomAD are based on) to those from RefSeq [22] (the basis for ClinVar).

### Data filtering

After merging the variations from the different sources, we applied filters to retain only: (i) variations that are caused by a single-nucleotide change and (ii) nonsense variations (Name column ending with “Ter” or “*”). This processing led to a total of 637 317 variations associated with 18 022 genes.

### Data annotation

The retained nonsense variations were processed to: (i) create a unique identifier corresponding to the HGVS (Human Genome Variation Society) standard [23], and (ii) merge the clinical status information into five unique categories: Pathogenic, Likely pathogenic, Uncertain significance, Likely benign, and Benign.

MAFs for different populations (worldwide, African, admixed American, Ashkenazi Jewish, East Asian, Finnish, Middle Eastern, non-Finnish European, South Asian, and Remaining) were obtained from gnomAD. For variations from gnomAD and COSMIC without pathogenicity status, we classified them as “Benign” if they had a worldwide MAF >0.005 [according to American College of Medical Genetics and Genomics (ACMG) guidelines] [24] and “Uncertain significance” otherwise. This led to the reclassification of 235 nonsense variations as “Benign.”

To obtain comprehensive protein and transcript information, we utilized the EDirect tool from the National Center for Biotechnology Information (NCBI). Specifically, we retrieved the total protein lengths corresponding to genes with nonsense variations using canonical protein identifiers. Identifying the position of the amino acid variation enabled us to calculate additional metrics, such as its relative position in relation to the total protein length. We also retrieved the Coding DNA Sequence (CDS) for each canonical transcript (MANE Select), to determine the PTC identity (UGA, UAG, or UAA) and the three nucleotides upstream and downstream of this codon.

From the RefSeq General Feature Format (GFF) file containing human genome annotations (GRCh38 assembly), we extracted information about the number of exons and their respective lengths for the canonical transcript. These data were crucial for assessing exon sensitivity to NMD according to the three NMD insensitivity zones: 150 nucleotides downstream of the start codon, 50 nucleotides upstream of the last exon–exon junction, and within the last exon.

By correlating the RefSeq canonical transcript identifier with its corresponding UniProt identifier [25], we also retrieved representative protein domains from the InterPro database [26].

To further characterize the nonsense variations, we extracted disease information from the Orphanet database (latest release, 2024–04) corresponding to a detailed description of the disease, clinical manifestations and symptoms, associated genes, and epidemiological data (prevalence) [27]. We identified 3206 diseases linked to genes with pathogenic nonsense variations. Finally, we retrieved the phenotypic description of 7765 phenotypes associated with the diseases from the Human Phenotype Ontology (HPO; v2024-04) [28].

### Database and web interface implementation

The architecture of StopKB is shown in Fig. 1b.

StopKB is a graph knowledgebase based on Neo4J (v4.4.3). Cypher is used as the query language for data import and retrieval processes, ensuring efficient management of the database. StopKB is structured around four primary types of nodes: nonsense variations, genes, diseases, and phenotypes. First, each nonsense variation node is linked to its corresponding gene node, characterizing the genetic basis of these variations. Second, the gene nodes are in turn connected to specific disease nodes to represent the genetic contributions to various medical conditions. Finally, the disease nodes are linked to their associated phenotype nodes, providing a comprehensive view of the clinical manifestations related to the genetic abnormalities. This graph-based structured approach allows an intuitive interpretation of the associations between genetic variations and their phenotypic outcomes.

To provide easy access *via* the web, we developed a user-friendly interface using Dash (version 2.7.0). Plotly (version 5.9.0) was also adopted to generate interactive charts. StopKB is freely available, and the content of the knowledgebase is available for download.

## Results

To enhance our understanding of the factors characterizing nonsense variations, we developed the StopKB knowledgebase. The database aggregates comprehensive information related to nonsense variations, including associated genes, resultant diseases, phenotypic manifestations as well as additional information/features that are either retrieved from existing databases or calculated in-house (nucleotide context, MAF, NMD sensitivity, etc.).

The public databases that provide the raw data for StopKB are subject to regular updates and continuous growth. Therefore, StopKB is updated monthly to ensure that it reflects the most current and comprehensive data available in the field. This frequent update process is vital to maintain the relevance and accuracy of the information provided.

### Overview of knowledgebase content

StopKB encompasses a comprehensive collection of nonsense variations, aggregating data from multiple reputable sources. Specifically, it contains 223 140 nonsense variations from COSMIC, 387 150 from gnomAD, and 64 348 from ClinVar. The relative overlap is illustrated with a Venn diagram in the Documentation section of the web interface. This results in a total of 637 317 unique nonsense variations. Among these, 45 255 are classified as “Pathogenic,” 9262 as “Likely pathogenic,” 582 189 as “Uncertain significance,” 274 as “Likely benign,” and 337 as “Benign.”

The nonsense variations are distributed across 18 022 genes. Of these genes, 3673 are implicated in 3206 diseases listed in Orphanet. Furthermore, these 3206 diseases have been associated with 7765 distinct phenotypes, providing a rich dataset for analysis.

The most crucial criterion to consider when prioritizing a nonsense variation is the PTC’s sensitivity to NMD as it provides information about the presence of mRNAs potentially coding for a truncated protein and/or accessible to nonsense suppression therapies. Considering the NMD insensitivity zones, we categorized 204 600 (∼32%) nonsense variations as NMD-insensitive. We also observed that 351 604 (∼55%) nonsense variations are located outside of any known protein domains as annotated in the InterPro database. Among these variations, 182 131 (∼29%) are found after the last representative domains in InterPro, thus leaving all domains of the proteins with these PTCs intact.

### Web user interface

**Figure 2.**
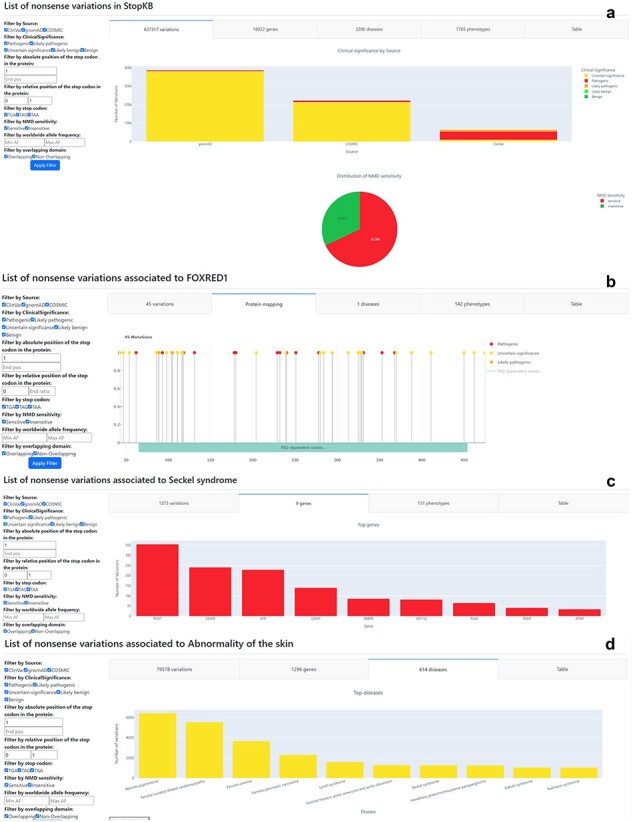
The “Search” results and Filter sections of the StopKB interface. Examples of StopKB outputs according to (a) database-wise query, (b) gene-wise query, (c) disease-wise query, and (d) phenotype-wise query. The user can dynamically filter the data using the filter panel on the left.

To facilitate the exploration of StopKB, we developed an interactive web interface (Fig. 2) with dynamic graphical visualizations. The web application enhances the utility of StopKB by facilitating data exploration at various levels of granularity and providing access to summary statistics.

Accessibility to the knowledgebase is ensured for different research uses. A dump of the knowledgebase is available for download on the “Download” page of the web interface. This allows researchers to execute intensive queries locally. The database is also available in the form of flat files. This dual availability—as a downloadable backup and in flat file format—ensures that researchers have flexible options for integrating the data in their own research environment and analyses. Finally, from the “Search” page of StopKB, the user can submit four types of queries: database-wise, gene-wise, disease-wise, and phenotype-wise. The results of queries can be further filtered based on several features, such as the database source of the variants, pathogenicity status, absolute or relative position of the amino acid variation within the protein sequence, PTC identity, sensitivity to NMD, allelic frequency, or presence within a protein domain.

### Database-wise query

The database-wise query (Fig. 2a) provides an overview of total counts of variants, genes, diseases, and phenotypes present in the current StopKB database. Upon submission with the keyword “StopKB,” it generates summary statistics encompassing the entire database. This query mode, complemented by dynamic filtering, enables users to explore the most common genes, diseases, and phenotypes within the knowledgebase. Additionally, users can quickly assess genes with the highest occurrences of NMD-insensitive variations.

### Gene-wise query

The primary advantage of the gene-wise query (Fig. 2b) lies in its ability to map variants to protein sequences. Upon selecting “Gene” and submitting a HGNC gene symbol, users are directed to a corresponding summary statistic page detailing all annotated nonsense variations within the specified gene. Users can explore gene-specific variant statistics, such as the number of clinical phenotypes linked to a particular gene or the distribution of pathogenic versus benign variants within the protein. For instance, querying “FOXRED1” reveals 45 nonsense variations associated with a single genetic disease. The interface displays variants mapped onto the protein sequence, accompanied by domain information sourced from InterPro. In regions with dense annotations or large proteins, users can zoom in on the protein sequence for enhanced visualization.

### Disease-wise query

By selecting the “Disease” query (Fig. 2c) and inputting a disease name, users are directed to a summary statistic page that outlines all annotated nonsense variations linked to the specified disease. Within the StopKB interface, users can address fundamental questions specific to the selected disease, such as determining the total number of genes or nonsense variations linked to a specific disorder. For instance, a disease query for “Seckel syndrome” highlights a total of 1273 nonsense variations distributed across 9 genes, and associated with 131 distinct phenotypes.

### Phenotype-wise query

The phenotype-wise query (Fig. 2d) allows users to explore annotated nonsense variations based on specific HPO terms. By selecting the “Phenotype” query and providing a phenotypic term, users are provided with a summary statistic page, presenting a detailed account of all annotated nonsense variations associated with the specified phenotype. The interface allows users to investigate phenotype-specific questions, such as identifying the predominant genes associated with the chosen phenotype. For example, searching for “Abnormality of the skin” reveals a diverse array of 79 578 nonsense variations dispersed across 1296 genes and 614 diseases.

### Example use case

**Figure 3.**
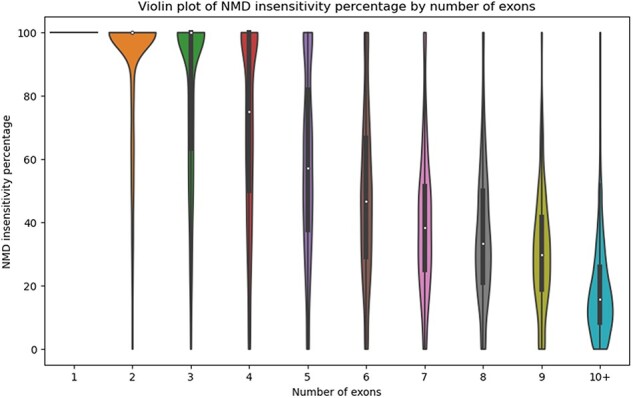
Violin plot of the percentage of NMD insensitivity according to the number of exons per gene.

There are numerous use cases for StopKB. For example, one might be interested in how the exon structure of an mRNA impacts NMD sensitivity. By grouping transcripts by the number of exons, we observed that the NMD insensitivity decreases as the number of exons increases (Fig. 3), consistent with what one might expect based on NMD insensitivity zones [12]. These results indicate that transcripts with a small number of exons are potential candidates for therapeutic approaches to restore a full-length functional protein.

## Discussion and future developments

The development of the StopKB knowledgebase represents a significant step towards consolidating and organizing information related to nonsense variations, genes, diseases, and phenotypes. By integrating data from multiple sources and providing a user-friendly interface, StopKB offers researchers and clinicians a valuable resource for exploring the genetic basis of diseases associated with PTCs. The monthly updates ensure that StopKB remains current and reflects the latest advancements in the field.

The extensive information contained in StopKB provides valuable insights into the landscape of nonsense variations and their implications. Notably, the categorization of nonsense variations based on their sensitivity to NMD is crucial for prioritizing targets for nonsense suppression therapies. The identification of NMD-insensitive variants, comprising approximately one-third of the dataset, suggests potential candidates for therapeutic intervention.

The interactive web interface of StopKB enhances its utility by providing researchers and clinicians with intuitive tools for data exploration. The ability to filter and extract specific data points based on various criteria facilitates targeted analyses. The database-wise, gene-wise, disease-wise, and phenotype-wise query functionalities enable users to navigate the vast knowledgebase efficiently in order to gain valuable insights into the relationships between genetic variations, diseases, and phenotypes.

The architecture and flexibility of StopKB is designed to enable its evolution beyond the initial application in genetic diseases. Although the knowledgebase is currently organized around four types of nodes: nonsense variations, genes, diseases, and phenotypes, a “Drugs” node could be defined to incorporate information on nonsense suppression therapies. Adding this type of data would not only allow for comparisons between different therapeutic approaches but also provide a framework for evaluating the efficacy of new molecules.

Moving forward, with the continual growth of genomic and clinical data, updating and expanding StopKB will be crucial. Integrating data on the efficacy of tested molecules, including outcomes from clinical trials and preclinical studies, would further enrich the knowledgebase. This should facilitate the discovery of new therapeutic targets and significantly contribute to personalized medicine by enabling researchers and clinicians to select the most promising treatments for specific patients based on their unique genetic profiles.

In conclusion, StopKB is more than just a collection of data on nonsense variations: it is an evolving platform that will adapt and grow with advances in research and gene therapy, offering exciting new perspectives in the field of personalized medicine.

## Data Availability

StopKB is available in the GitHub repository https://github.com/Dichopsis/StopKB.
